# Comparative mitochondrial genome analysis of *Daphnis nerii* and other lepidopteran insects reveals conserved mitochondrial genome organization and phylogenetic relationships

**DOI:** 10.1371/journal.pone.0178773

**Published:** 2017-06-09

**Authors:** Yu Sun, Chen Chen, Jin Gao, Muhammad Nadeem Abbas, Saima Kausar, Cen Qian, Lei Wang, Guoqing Wei, Bao-Jian Zhu, Chao-Liang Liu

**Affiliations:** College of Life Sciences, Anhui Agricultural University, Hefei, China; Institute of Plant Physiology and Ecology Shanghai Institutes for Biological Sciences, CHINA

## Abstract

In the present study, the complete sequence of the mitochondrial genome (mitogenome) of *Daphnis nerii* (Lepidoptera: Sphingidae) is described. The mitogenome (15,247 bp) of *D*.*nerii* encodes13 protein-coding genes (PCGs), 22 transfer RNA genes (tRNAs), two ribosomal RNA genes (rRNAs) and an adenine (A) + thymine (T)-rich region. Its gene complement and order is similar to that of other sequenced lepidopterans. The 12 PCGs initiated by ATN codons except for cytochrome c oxidase subunit 1 (*cox1*) gene that is seemingly initiated by the CGA codon as documented in other insect mitogenomes. Four of the 13 PCGs have the incomplete termination codon T, while the remainder terminated with the canonical stop codon. This mitogenome has six major intergenic spacers, with the exception of A+T-rich region, spanning at least 10 bp. The A+T-rich region is 351 bp long, and contains some conserved regions, including ‘ATAGA’ motif followed by a 17 bp poly-T stretch, a microsatellite-like element (AT)_9_ and also a poly-A element. Phylogenetic analyses based on 13 PCGs using maximum likelihood (ML) and Bayesian inference (BI) revealed that *D*. *nerii* resides in the Sphingidae family.

## 1. Background

The oleander hawk moth, *D*.*nerii* (Lepidoptera: Sphingidae) is one of the most widely distributed species of Sphingidae. It occursin the tropical and subtropical regions ranging from Africa to south-east Asia. It was first reported on Guam in August, 2005 as a plant pest. It feeds on a variety of plant species ranging from shrubs to trees such as *Catharanthus*, *Vinca*, *Adenium*, *Vitis*, *Tabernaemontana*, *Gardenia*, *Trachelospermum*, *Amsonia*, *Asclepias*, *Carissa*, *Rhazya*, *Thevetia*, *Jasminum* and *Ipomoea*. While, *Nerium oleander* has been documented as the most preferred host of the *D*.*nerii*. The management of this species is extremely important and that require deep knowledge on its different biological aspects[1]. Although a few studies are available on its ecology, reproduction and development and so on but its genetic characteristics are rarely documented. To improve the management of the *D*.*nerii*, it is extremely important to know more knowledge about this pest, particularly its genetic characteristics and phylogentic position. Moreover, the study of mitogenome is an important subject to understand molecular evolution, comparative and evolutionary genomics, phylogenetics, and population genetics [2–4].

The metazoan mitogenome is a closed-circular DNA molecule, ranged in size from 14 to 19 kilobases (kb), including intergenic spacers being very short or absent[5]. It contains 13 protein-coding genes (PCGs), 2 ribosomal RNA genes (rRNAs), and 22 transfer RNA genes (tRNAs)[6]. In addition, there is one major non-coding region (control region) that in other Lepidopterans and in invertebrates is named as A+T-rich region because of its enormously high content in Adenines and Thymines. This control region is generally believed to control the initiation of transcription and replication of animal mitogenome[7].

The order Lepidoptera is one of the largest insect orders and includes greater than 160 000 described species that are classified into 45–48 superfamilies[8]. Sphingidae is one of the most diverse superfamilies, and contains 203 genera and 1348 species distributed worldwide. Despite this enormous species diversity, only two complete mitogenomes are available in GenBank (Table 1)[9]. Newly accessible Lepidoptera mitogenomes will provide further insight into our understanding of evolutionary relationships between these species. In this study, we described the complete sequence of the mitogenome of *D*. *nerii* and compared it with other Lepidoptera species sequenced to date to highlight evolution of Lepidopterans, particularly, phylogenetic relation-ships of Bombycoidea.

**Table 1:** Details of the lepidopteran mitogenomes used in this study.

Superfamily	Family	Species	Size (bp)	GenBank accession no.	Reference
Bombycoidea	Bombycidae	*Bombyx mandarina*	15,682	NC_003395	[33]
		*Bombyx mori*	15,643	NC_002355	Direct submission
	Saturniidae	*Actias selene*	15,236	NC_018133	[34]
		*Eriogyna pyretorum*	15,327	NC_012727.1	[5]
		*Antheraea pernyi*	15,566	AY242996	[35]
		*Antheraea yamamai*	15,338	NC_012739	[36]
	Sphingidae	*Manduca sexta*	15,516	NC_010266	[9]
		*Sphinx morio*	15,299	NC_020780.1	[37]
		*Notonagemia analis scribae*	15,303	KU934302.1	[38]
		***Daphnis nerii***	15,247		**This study**
Noctuoidea	Lymantriidae	*Lymantria dispar*	15,569	NC_012893	Unpublished
		*Amata formosae*	15,463	KC513737	[6]
		*Hyphantria cunea*	15,481	NC_014058	[28]
	Noctuidae	*Agrotis ipsilon*	15,377	KF163965	[39]
Geometroidea	Geometridae	*Apocheima cinerarium*	15,722	KF836545	[40]
		*Biston panterinaria*	15,517	NC_020004	[41]
		*Phthonandria atrilineata*	15,499	NC_010522	[27]
		*Biston thibetaria*	15,484	KJ670146.1	Unpublished
		*Biston suppressaria*	15,628	KP278206	[42]
		*Jankowskia athleta*	15,534	KR822683	[43]
Pyraloidea	Crambidae	*Chilo suppressalis*	15,395	NC_015612	[32]
		*Elophila interruptalis*	15,351	NC_021756	[44]
		*Diatraea saccharalis*	15,490	NC_013274	[45]
	Pyralidae	*Corcyra cephalonica*	15,273	NC_016866.1	[46]
Gelechioidea	Elachistidae	*Promalactis suzukiella*	15,507	NC_026697	[47]
Tortricoidea	Tortricidae	*Acleris fimbriana*	15,933	NC_018754	Unpublished
		*Adoxophyes orana*	15,343	JX872403	[48]
Papilionoidea	Papilionidae	*Parnassius bremeri*	15,389	NC_014053	[49]
		*Papilio syfanius*	15,359	NC_023978	[50]
		*Papilio maraho*	16,094	NC_014055	[29]
		*Teinopalpus aureus*	15,242	NC_014398	Unpublished
Yponomeutoidea	Plutellidae	*Plutella xylostella*	16,179	JF911819	[51]
	Lyonetiidae	*Leucoptera malifoliella*	15,646	NC_018547	[52]
Hepialoidea	Hepialidae	*Thitarodes renzhiensis*	16,173	NC_018094	[53]
		*Ahamus yunnanensis*	15,816	NC_018095	[53]
		*Thitarodes pui*	15,064	NC_023530	[54]

## 2. Materials and methods

### 2.1 Experimental insects and DNA extraction

The *D*. *nerii* specimens were collected from Anhui Agricultural University, Anhui Province, China. The total DNA was extracted using the Genomic DNA Extraction Kit, according to the manufacturer's instructions (Aidlab Co., Beijing, China). The extracted DNA quality was examined by 1% agarose gel electrophoresis (w/v) and used to amplify the complete mitogenome of *D*. *nerii*.

### 2.2 PCR amplification and sequencing

We designed twelve pairs of primers from the conserved nucleotide sequences of known mitogenome of Lepidopteran species to determine the *D*. *nerii* mitogenome[10, 11]. The complete list of successful primer is given in Table 2 (Sangon Biotech Co., Shanghai, China). All amplifications were performed on an Eppendorf Mastercycler and Mastercycler gradient in 50μL reaction volumes, which contained 35μL sterilized distilled water, 5μL 10×Taq buffer (Mg^2+^ plus), 4 μL dNTP (25 mM), 1.5 μL extracted DNA as template, forward and reverse primers 2 μL each (10 μM) and 0.5 μL (1 unit) TaqDNA polymerase (Takara Co., Dalian, China). The PCR amplification conditions were as follows: an initial denaturation one cycle at 94°C for 4 min followed by 38 cycles, one cycle at 94°C for 30 s, one cycle at 48–59°C for 1–3 min (depending on the putative length of the fragments), and a final extension one cycle at 72°C for 10 min. The PCR products were detected by 1% agarose gel electrophoresis (w/v), and were purified using a DNA gel extraction kit (Transgen Co., Beijing, China), and directly sequenced with PCR primers.

**Table 2:** Details of the primers used to amplify the mitogenome of *D*. *nerii*.

Primer pair	Primer sequences (5’-3’)
**F1**	TAAAAATAAGCTAAATTTAAGCTT
**R1**	TATTAAAATTGCAAATTTTAAGGA
**F2**	AAACTAATAATCTTCAAAATTAT
**R2**	AAAATAATTTGTTCTATTAAAG
**F3**	TGGAGCAGGAACAGGATGAAC
**R3**	GAGACCADTACTTGCTTTCAG
**F4**	ATTTGTGGAGCTAATCATAG
**R4**	GGTCAGGGACTATAATCTAC
**F5**	TCGACCTGGAACTTTAGC
**R5**	GCAGCTATAGCCGCTCCTACT
**F6**	TAAGCTGCTAACTTAATTTTTAGT
**R6**	CCTGTTTCAGCTTTAGTTCATTC
**F7**	CCTAATTGTCTTAAAGTAGATAA
**R7**	TGCTTATTCTTCTGTAGCTCATAT
**F8**	TAATGTATAATCTTCGTCTATGTAA
**R8**	ATCAATAATCTCCAAAATTATTAT
**F9**	ACTTTAAAAACTTCAAAGAAAAA
**R9**	TCATAATAAATTCCTCGTCCAATAT
**F10**	GGAGCTTCTACATGAGCTTTTGG
**R10**	GTTTGCGACCTCGATGTTG
**F11**	GGTCCCTTACGAATTTGAATATATCCT
**R11**	AAACTAGGATTAGATACCCTATTAT
**F12**	CTCTACTTTGTTACGACTTATT
**R12**	TCTAGGCCAATTCAACAACC

### 2.3 Sequence assembly and gene annotation

Sequence annotation was performed using blast tools available from the NCBI (https://blast.ncbi.nlm.nih.gov/Blast.cgi), and SeqMan II program from the Lasergene software package (DNASTAR Inc.; Madison, USA). The protein-coding sequences were translated into putative proteins on the basis of the Invertebrate Mitochondrial Genetic Code. The skewness was measured by the method given by Junqueiraet al.[12], and the base composition of nucleotide sequences were described as: AT skew = [A−T]/[A+T], GC skew = [G−C]/[G+C]. The relative synonymous codon usage (RSCU) values were calculated using MEGA 5.1[13].

The tRNA genes were determined using the tRNAscan-SE software (http://lowelab.ucsc.edu/tRNAscan-SE/) [14], or predicted by sequence features of being capable of folding into the typical cloverleaf secondary structure with legitimate anticodon. The tandem repeats in the A+T-rich region were determined by the tandem repeats finder program (http://tandem.bu.edu/trf/trf.html)[15].

### 2.4 Phylogenetic analysis

To reconstruct the phylogenetic relationship among Lepidopterans, 36 complete or near-complete mitogenomes were downloaded from the GenBank database (Table 1). The mitogenomes of *Drosophila melanogaster* (U37541.1)[16] and *Locusta migratoria* (NC_001712)[17] were used as outgroup. The multiple alignments of the 13 PCGs concatenated nucleotide sequences were conducted using ClustalX version 2.0.[18]. Then concatenated set of nucleotide sequences from the 13 PCGs was used for phylogenetic analyses, which were performed using Maximum Likelihood (ML) method with the MEGA version 5.1 program[13] and Bayesian Inference (BI) with MrBayes 3.2 version program[19]. The ML analyses were used to infer phylogenetic trees with 1000 bootstrap replicates. BI analysis as the following conditions: the Markov chains were run for 100,000 generations with trees being sampled every 100 generations. The consensus trees were visualized by FigTree v1.4.2 (http://tree.bio.ed.ac.uk/software/figtree/) program with adjustable settings.

## 3. Results and discussion

### 3.1 Genome structure, organization and composition

**Fig 1:**
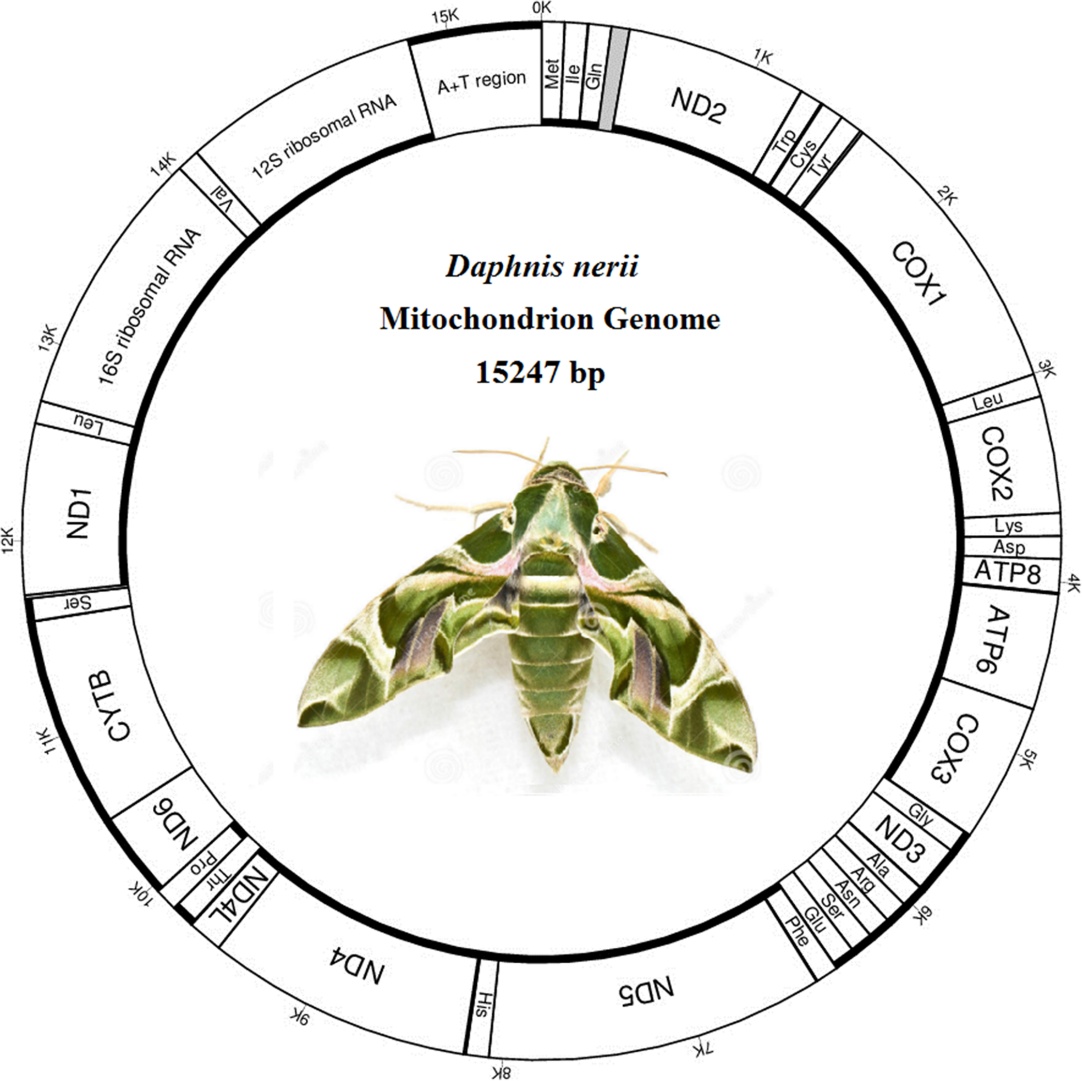
Map of the mitogenome of *D*.*nerii*. The tRNA genes are labeled according to the IUPAC-IUB single-letter amino acids: *cox1*, *cox2* and *cox3* refer to the cytochrome c oxidase subunits; *cob* refers to cytochrome b; *nad1*-*nad6* refer to NADH dehydrogenase components; *rrnL* and *rrnS* refer to ribosomal RNAs.

The complete sequence of the mitogenome of *D*.*nerii* is 15,247 bp in length (S1 File and Fig 1), which is well within the range observed in the whole sequenced Lepidoptera species with the size ranging from 15,682 bp in *Bombyx mandarina* (Bombycidae) to 15,064bp in *Thitarodespui* (Hepialidae) (Table 1). Alignment with previously sequenced lepidopteran mitogenomes revealed 38 mitogenome regions, including 13 protein-encoding regions (PCGs: *atp6*, *atp8*, *cox1*, *cox2*, *cox3*, *cytb*, *nad1*, *nad2*, *nad3*, *nad4*, *nad5*, *nad6*, *and nad4L*), two rRNA-encoding regions (large and small ribosomal RNA), 22 tRNA-encoding regions (transfer RNA) and a large non-coding-region with high A+T-rich composition that is usually found in most animal mtDNAs (Table 3). The gene arrangement and orientation of *D*.*nerii* mitogenome is *trnM*-*trnI*-*trnQ* that is different from the ancestral gene order *trnI*-*trnQ*-*trnM*[2].

**Table 3:** List of annotated mitochondrial genes of *D*. *nerii*.

Gene	Direction	Location	Size	Anti codon	Start codon	Stop codon	Intergenic Nucleotides
tRNA^Met^	F	1–68	68	CAT	—	—	0
tRNA^Ile^	F	69–136	68	GAT	—	—	-3
tRNA^Gln^	R	134–202	69	TTG	—	—	55
nad2	F	256–1273	1018		ATT	TAA	-2
tRNA^Trp^	F	1272–1341	70	TCA	—	—	2
tRNA^Cys^	R	1334–1397	64	GCA	—	—	0
tRNA^Tyr^	R	1398–1461	64	GTA	—	—	14
cox1	F	1476–3004	1529		CCA	T	0
tRNA^Leu(UUR)^	F	3005–3071	67	TAA	—	—	0
cox2	F	3072–3753	682		ATG	T	0
tRNA^Lys^	F	3754–3824	71	CTT	—	—	1
tRNA^Asp^	F	3826–3892	67	GTC	—	—	0
atp8	F	3893–4057	165		ATC	TAA	-7
atp6	F	4051–4728	678		ATG	TAA	-1
cox3	F	4728–5524	795		ATG	TAA	2
tRNA^Gly^	F	5527–5595	69	TCC	—	—	0
nad3	F	5596–5948	353		ATC	TAA	3
tRNA^Ala^	F	5952–6017	66	TGC	—	—	-1
tRNA^Arg^	F	6017–6081	65	TCG	—	—	0
tRNA^Asn^	F	6082–6148	67	GTT	—	—	-1
tRNA^Ser(AGN)^	F	6148–6214	67	GCT	—	—	-1
tRNA^Glu^	F	6214–6279	66	TTC	—	—	-2
tRNA^Phe^	R	6278–6344	67	GAA	—	—	0
nad5	R	6345–8067	1723		ATA	T	0
tRNA^His^	R	8083–8139	57	GTG	—	—	24
nad4	R	8150–9537	1388		ATT	T	-1
nad4L	R	9537–9821	285		ATG	TAA	4
tRNA^Thr^	F	9826–9890	65	TGT	—	—	-1
tRNA^Pro^	R	9890–9955	66	TGG	—	—	1
nad6	F	9957–10488	532		ATG	TAA	-1
cytb	F	10488–11630	1143		ATG	TAA	-6
tRNA^Ser(UCN)^	F	11625–11689	65	TGA	—	—	18
nad1	R	11708–12644	937		ATG	TAA	0
tRNA^Leu(CUN)^	R	12645–12711	67	TAG	—	—	0
rrnL	R	12712–14049	1338	—	—	—	0
tRNA^Val^	R	14050–14115	66	TAC	—	—	1
rrnS	R	14117–14895	778	—	—	—	1
A+T-rich Region		14897–15247	351				

The comparison of *D*. *nerii* mitogenome composition and skewness level with other sequenced Lepidoptera species is represented in Table 4. The genome composition of the major strand is A: 40.81%, T: 39.48%, G: 7.58%, and C: 12.13%, with a total A+T content of 80.29%. Additionally, it exhibits positive AT skewness (0.017) and negative GC skewness (-0.231). The AT-skewness in other Lepidopteran mitogenomes sequenced to date, ranges from 0.057 (*B*. *mandarina*) to -0.027 (*A*. *formosae*), while the GC-skewness from -0.266 (*A*. *formosae*) to -0.174 (*G*. *argentata*). Moreover the positive AT skewness (0.017) indicates the occurrence of more As than Ts that has also been reported in several other lepidopteran species such as *B*. *mandarina* (0.057), *H*. *cunea* (0.010) and *L*. *dispar* (0.016).

**Table 4:** Composition and skewness in different Lepidopteran mitogenomes.

Species	Size(bp)	A%	G%	T%	C%	A+T%	ATskewness	GCskewness
**Whole genome**								
***D*. *nerii***	15,247	40.81	7.58	39.48	12.13	80.29	0.017	-0.231
*M*. *sexta*	15,516	40.67	7.46	41.11	10.76	81.79	-0.005	-0.181
*S*. *morio*	15,299	40.64	7.58	40.53	11.23	81.17	0.001	-0.194
*B*. *mandarina*	15,682	43.11	7.40	38.48	11.01	81.59	0.057	-0.196
*A*. *pernyi*	15,566	39.22	7.77	40.94	12.07	80.16	-0.021	-0.216
*L*. *dispar*	15,569	40.58	7.57	39.30	12.55	79.88	0.016	-0.248
*L*. *melli*	15,418	39.38	8.72	39.29	13.06	78.67	0.001	-0.199
*H*. *cunea*	15,481	40.58	7.55	39.81	12.06	80.39	0.010	-0.230
*A*. *formosae*	15,463	38.67	7.53	40.83	12.98	79.49	-0.027	-0.266
*G*. *argentata*	15,337	39.64	7.56	42.05	10.75	81.69	0.030	-0.174
*C*. *pomonella*	15,253	39.92	7.88	40.21	11.99	80.13	-0.004	-0.207
*P*.*atrilineata*	15,499	40.78	7.67	40.24	11.31	81.02	0.007	-0.192
*A*. *ilia*	15,242	39.77	7.75	40.68	11.80	80.45	-0.011	-0.207
*G*. *dimorpha*	15,831	39.99	7.77	40.85	11.39	80.84	-0.011	-0.189
*H*. *vitta*	15,282	39.58	7.81	40.34	12.27	79.92	-0.010	-0.222
*C*. *suppressalis*	15,395	40.64	7.39	40.03	11.94	80.67	0.007	-0.235
*A*. *ipsilon*	15,377	40.38	7.71	40.87	11.04	81.25	-0.006	-0.178
**PCG**								
***D*. *nerii***	11,208	40.52	8.32	38.15	13.00	78.68	0.030	-0.220
*M*. *sexta*	11,185	40.41	8.23	39.88	11.48	80.30	0.007	-0.165
*S*. *morio*	11,179	40.28	8.27	39.56	11.89	79.84	0.009	-0.180
*B*. *mandarina*	11,196	42.83	8.26	37.04	11.87	79.87	0.072	-0.179
*A*. *pernyi*	11,204	39.22	7.77	40.94	12.07	80.16	-0.021	-0.216
*L*. *dispar*	11,227	39.67	8.44	38.16	13.73	77.83	0.019	-0.239
*L*. *melli*	11,120	38.47	9.17	38.17	14.19	76.64	0.004	-0.215
*H*. *cunea*	11,198	39.98	8.35	38.61	13.06	78.59	0.017	-0.220
*A*. *formosae*	11,217	38.18	8.28	39.62	13.92	77.80	-0.019	-0.254
*G*. *argentata*	10,303	38.10	8.61	41.88	11.41	79.98	-0.047	-0.140
*C*. *pomonella*	11,199	39.55	8.69	39.00	12.76	78.55	0.007	-0.190
*P*.*atrilineata*	11,203	40.23	8.59	38.87	12.31	79.10	0.017	-0.178
*A*. *ilia*	11,148	39.41	8.41	39.49	12.69	78.89	-0.001	-0.203
*G*. *dimorpha*	11,232	39.51	8.81	39.18	12.49	78.69	0.004	-0.173
*H*. *vitta*	11,202	38.76	8.61	39.43	13.20	78.19	-0.009	-0.210
*C*. *suppressalis*	11,230	40.42	8.16	38.48	12.95	78.90	0.025	-0.227
*A*. *ipsilon*	11,226	39.69	8.44	40.14	11.72	79.83	-0.006	-0.163
**tRNA**								
***D*. *nerii***	1,586	41.74	7.38	40.79	10.09	82.53	0.012	-0.155
*M*. *sexta*	1,554	40.99	7.92	41.06	10.04	82.05	-0.001	-0.118
*S*. *morio*	1,462	40.63	8.21	40.97	10.19	81.60	-0.004	-0.107
*B*. *mandarina*	1,472	41.78	7.81	39.95	10.46	81.73	0.022	-0.145
*A*. *pernyi*	1,459	39.22	7.77	40.94	12.07	80.16	-0.021	-0.217
*L*. *dispar*	1,459	41.60	7.95	39.48	10.97	81.08	0.026	-0.160
*L*. *melli*	1,486	40.58	8.55	40.24	10.63	80.82	0.004	-0.109
*H*. *cunea*	1,463	41.83	7.86	39.99	10.32	81.82	0.022	-0.135
*A*. *formosae*	1,457	40.43	7.96	40.36	11.26	80.78	0.001	-0.172
*G*. *argentata*	1,468	41.35	8.24	40.19	10.22	81.54	0.014	-0.107
*C*. *pomonella*	1,464	41.19	7.92	40.23	10.66	81.42	0.012	-0.147
*P*.*atrilineata*	1,476	41.4	8.2	40.04	10.37	81.44	0.017	-0.117
*A*. *ilia*	1,433	40.61	8.30	40.96	10.12	81.58	-0.004	-0.099
*G*. *dimorpha*	1,451	41.01	8.06	40.52	10.41	81.53	0.006	-0.127
*H*. *vitta*	1,456	41.41	8.04	39.84	10.71	81.25	0.019	-0.142
*C*. *suppressalis*	1,482	40.89	7.89	40.89	10.32	81.78	0.000	-0.133
*A*. *ipsilon*	1,465	41.23	8.12	40.48	10.17	81.71	0.009	-0.112
**rRNA**								
***D*. *nerii***	2,117	42.14	4.87	42.61	10.39	84.74	-0.006	-0.362
*M*. *sexta*	2,168	41.37	4.84	44.05	9.73	85.42	-0.031	-0.336
*S*. *morio*	2,152	41.73	4.83	43.08	10.36	84.8	-0.016	-0.364
*B*. *mandarina*	2,134	43.86	4.78	41.05	10.31	84.91	0.033	-0.366
*A*. *pernyi*	2,144	39.22	7.77	40.94	12.07	80.16	-0.021	-0.217
*L*. *dispar*	2,150	42.79	4.79	41.81	10.60	84.60	0.012	-0.377
*L*. *melli*	2,233	42.23	4.93	41.96	10.88	84.19	0.003	-0.376
*H*. *cunea*	2,234	42.08	4.92	42.75	10.25	84.83	-0.008	-0.351
*A*. *formosae*	2,163	38.93	4.72	44.85	11.51	83.77	-0.071	-0.418
*G*. *argentata*	2,165	40.6	4.76	45.13	9.52	85.73	-0.053	-0.333
*C*. *pomonella*	2,147	40.48	5.03	43.92	10.57	84.4	-0.041	-0.355
*P*.*atrilineata*	2,203	42.85	4.58	43.08	9.49	85.93	-0.003	-0.349
*A*. *ilia*	2,109	40.11	4.98	44.86	10.05	84.97	-0.056	-0.337
*G*. *dimorpha*	2,181	41.13	4.95	43.83	10.09	84.96	-0.032	-0.342
*H*. *vitta*	2,194	41.43	4.88	43.25	10.44	84.69	-0.021	-0.363
*C*. *suppressalis*	2,171	41.27	4.97	43.67	10.09	84.94	-0.028	-0.340
*A*. *ipsilon*	2,162	41.58	5	43.57	9.85	85.15	-0.023	-0.327
**A+T-rich region**								
***D*. *nerii***	351	41.60	1.42	53.56	3.42	95.16	-0.126	-0.413
*M*. *sexta*	324	45.06	1.54	50.31	3.09	95.37	-0.005	-0.335
*S*. *morio*	316	44.3	2.53	48.42	4.75	92.72	-0.044	-0.305
*B*. *mandarina*	484	46.49	2.69	47.93	2.89	94.42	-0.015	-0.036
*A*. *pernyi*	552	39.22	7.77	40.94	12.07	80.16	-0.021	-0.216
*L*. *dispar*	435	40.58	7.57	39.30	12.55	79.88	0.016	-0.248
*L*. *melli*	338	43.2	1.48	51.18	4.14	94.38	-0.085	-0.473
*H*. *cunea*	357	45.66	1.12	49.3	3.92	94.96	-0.038	-0.556
*A*. *formosae*	482	42.95	2.9	49.79	4.36	92.74	-0.074	-0.201
*G*. *argentata*	340	43.24	1.47	52.06	3.24	95.29	-0.093	-0.376
*C*. *pomonella*	351	43.3	1.14	52.42	3.13	95.73	-0.095	-0.466
*P*.*atrilineata*	457	40.7	0.66	57.55	1.09	98.25	-0.172	-0.246
*A*. *ilia*	403	42.93	3.23	49.63	4.22	92.56	-0.072	-0.133
*G*. *dimorpha*	848	41.63	1.30	54.83	2.24	96.46	-0.137	-0.266
*C*. *suppressalis*	348	42.24	0.29	53.16	4.31	95.4	-0.114	-0.874
*A*. *ipsilon*	332	46.08	1.51	48.8	3.61	94.88	-0.029	-0.41

### 3.2 Protein-coding genes and codon usage

The mitogenome of *D*.*nerii* contains 13 protein-coding genes. Most protein-coding genes (12 PCGs) begin with ATN (one with ATA, two with ATT, seven with ATG and two with ATC) codons, except for the *cox1*. The *cox1* gene of *D*.*nerii* seems to be started with CCA codon as previously documented in *Cerura menciana*[20] and in *Spoladea recurvalis*[21]. Several authors have maintained the problematic translational start at the *cox1* locus in many insect species, with TTAG, ACG, and TTG proposed as start codons for *cox1*[22–24]. A most common stop codon of the PCGs is TAA, but an incomplete termination stop codon T is present at *cox1*, *cox2*, *nad5* and *nad4*. This has been well documented in other invertebrate mitogenomes and is a common evolutionary feature shared by mtDNA. The single T stop codon was recognized by endonucleases processing the polycistronic pre-mRNA transcription, and produced functional stop codons by polyadenylation from its contiguous PCGs[25].

**Fig 2:**
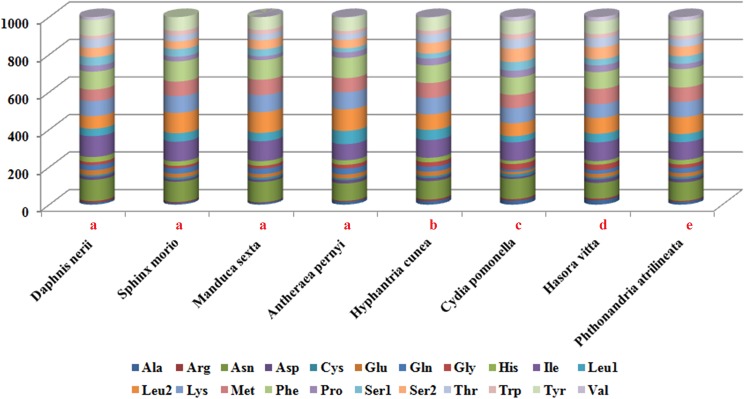
Comparison of codon usage within the mitochondrial genome of members of the Lepidoptera. Lowercase letters (a, b, c, d and e) above species name represent the superfamily to which the species belongs (a: Bombycoidea, b: Noctuoidea, c: Tortricoidea, d: Hesperioidea, e: Geometroidea).

**Fig 3:**
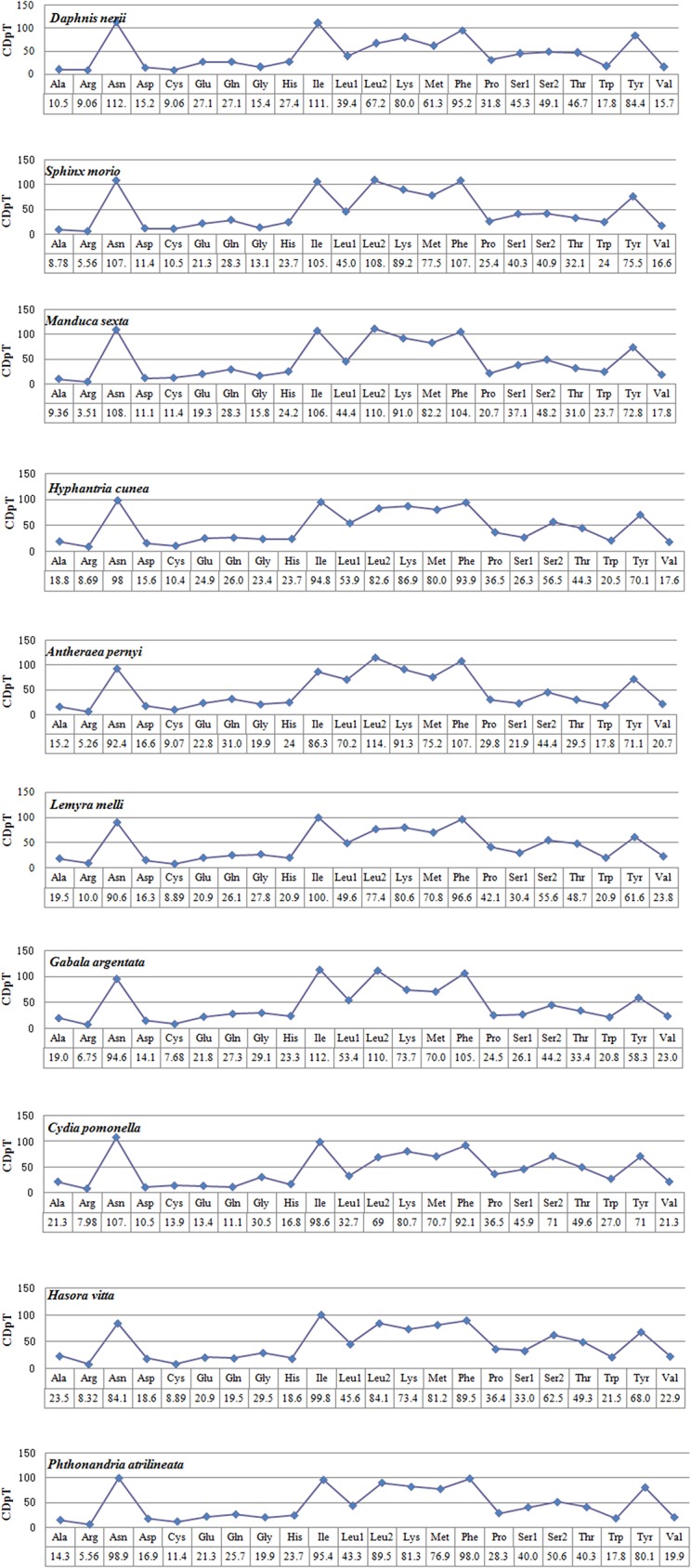
Codon distribution in members of the Lepidoptera.CDspT = codons per thousand codons.

We analyzed the codon usage among eight Lepidopteran species, of which four belong to Bombycoidea and one each from Noctuoidea, Tortricoidea, Hesperioidea and Geometroidea (Fig 2). The results revealed that Asn, Ile, Leu2, Lys, Phe, Tyr and Met were the most frequently utilized amino acids. There were at least 4 codon families with no less than 60 codons per thousand codons (Leu2, Lys, Met, and Tyr), and 3 families with at least 80 codons per thousand codons (Asn, Ile and Phe) that were observed in the 8 insect species. The rarest used codon family was Arg. Codon distributions of four species in Bombycoidea are in consistency, and each amino acid has equal contents in different species (Fig 3).

**Fig 4:**
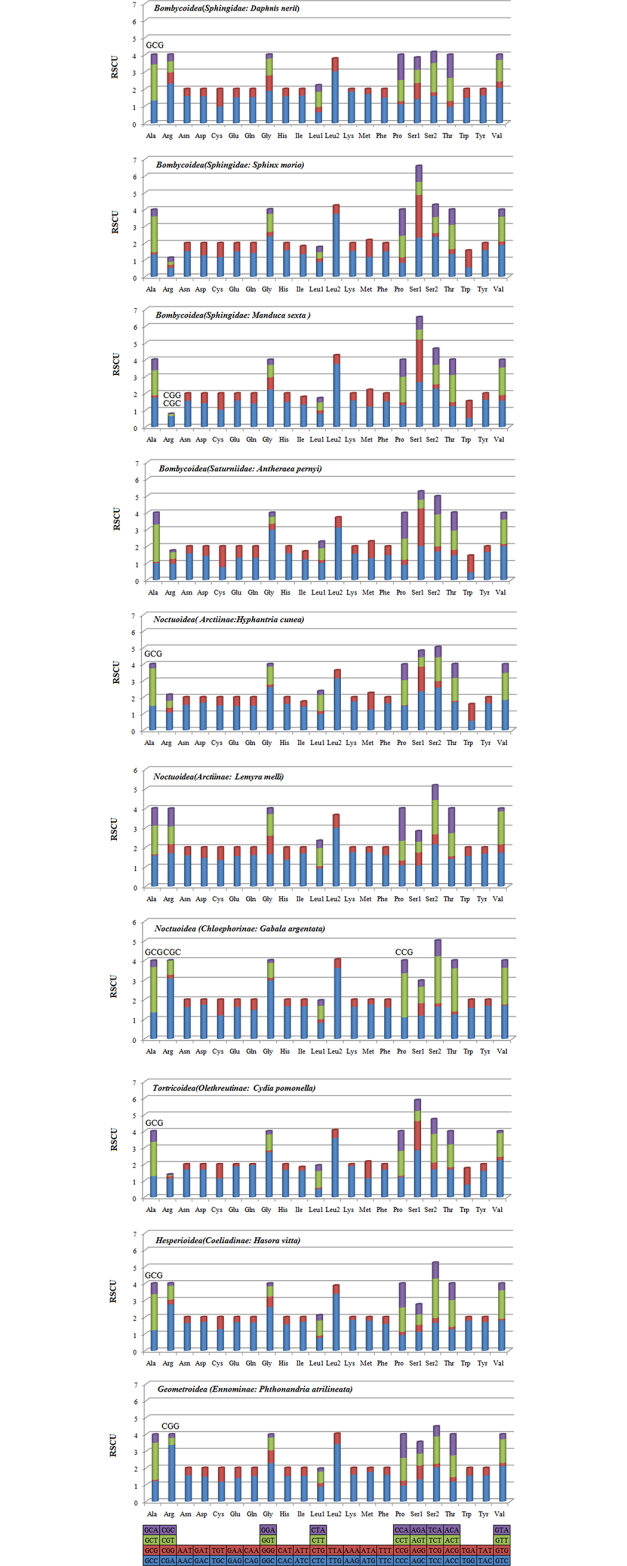
The Relative Synonymous Codon Usage (RSCU) of the mitochondrial genome of six superfamilies in the Lepidoptera. Codon families are plotted on the X axis. Codons indicated above the bar are not present in the mitogenome.

The Relative Synonymous Codon Usage (RSCU) was assessed in the PCGs for five available Lepidopteran superfamilies mitogenomes (Fig 4). All possible codon combinations are present in the PCGs of *D*.*nerii* except for the GCG. The absence of codons containing high GC content is also a characteristic feature of several Lepidopteran species such as *M*. *sexta* (CGG&CGC), *H*. *cunea*(GCG), *G*. *argentata* (GCG&CGC&CCG), *P*. *atrilineata* (CGG), *C*. *pomonella* (GCG), *H*. *vitta* (GCG), and so on. Further, these codons are likely to be less, and this featureis conserved in insect mitogenomes[20, 26].

### 3.3 Ribosomal and transfer RNA genes

The mitogenome of *D*.*nerii* includes two rRNA genes usually present in other animals sequenced to date. The large ribosomal gene (*rrnL*) is 1338 bp long, and resided between tRNA Leu (CUN) and tRNA Val, whereas the small ribosomal gene (*rrnS*) is only 778 bp long, and located between tRNA Val and A+T-rich region (Table 3). The A+T content (84.74%) of two rRNAs fall within the range from 80.16% (*A*.*pernyi*) to 85.93% (*P*. *atrilineata*) of Lepidopterans. Both AT skewness (-0.006) and GC skewness (-0.362) are negative, that is similar to other previously sequenced Lepidopteran mitogenome[6, 27].

**Fig 5:**
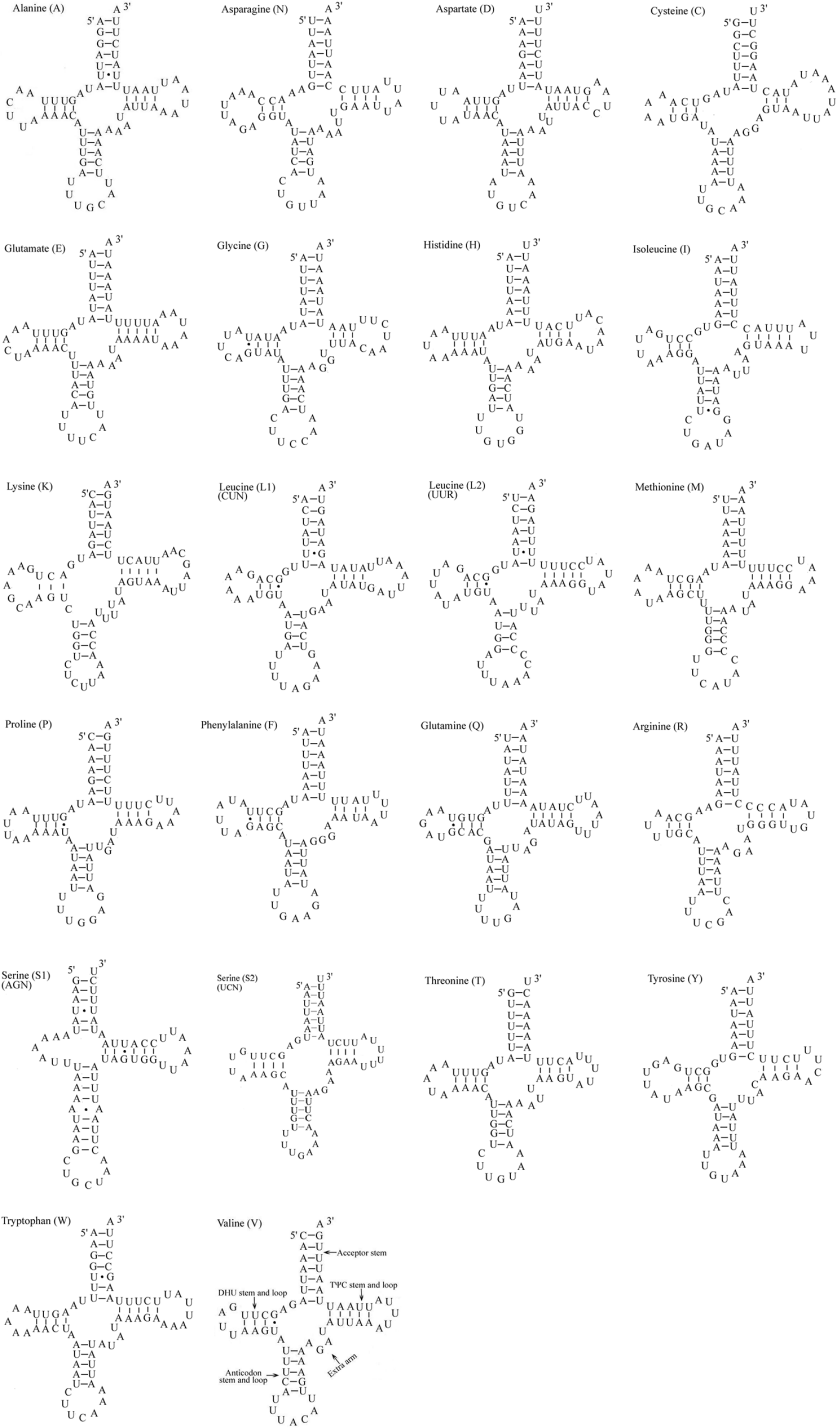
Putative secondary structures of the 22 tRNA genes of the *D*.*nerii* mitogenome.

The *D*.*nerii* harbors an entire set of 22 tRNA (ranging from 64 to 71 nucleotides in length) commonly present in most of Lepidopteran mitogenomes. This region is highly A+T biased, accounting for 82.53%, and exhibit positive AT-skewness (0.012), while negative GC skewness (-0.155) (Table 4). All tRNA spresented the typical cloverleaf secondary structure but *trnS1* lacked the DHU stem (Fig 5) similar to several other previously sequenced Lepidopterans[10, 28]. Moreover 14 of the 22 tRNA genes were coded by the H-strand and remainder eight by the L-strand.

A total of 15 mismatched bps in the *D*.*nerii* tRNAs were identified. Most of them are G-U wobble pairs scatter throughout ten tRNAs (two in acceptor stem, seven in DHU, one in anticodon stem, and one in TψC), a A-A mismatch in the anticodon stem of the *trnS1* and three U-U mismatches in acceptor stem of the *trnA*, *trnL2* and *trnS1* (Fig 5).

### 3.4 Overlapping and intergenic spacer regions

**Fig 6:**
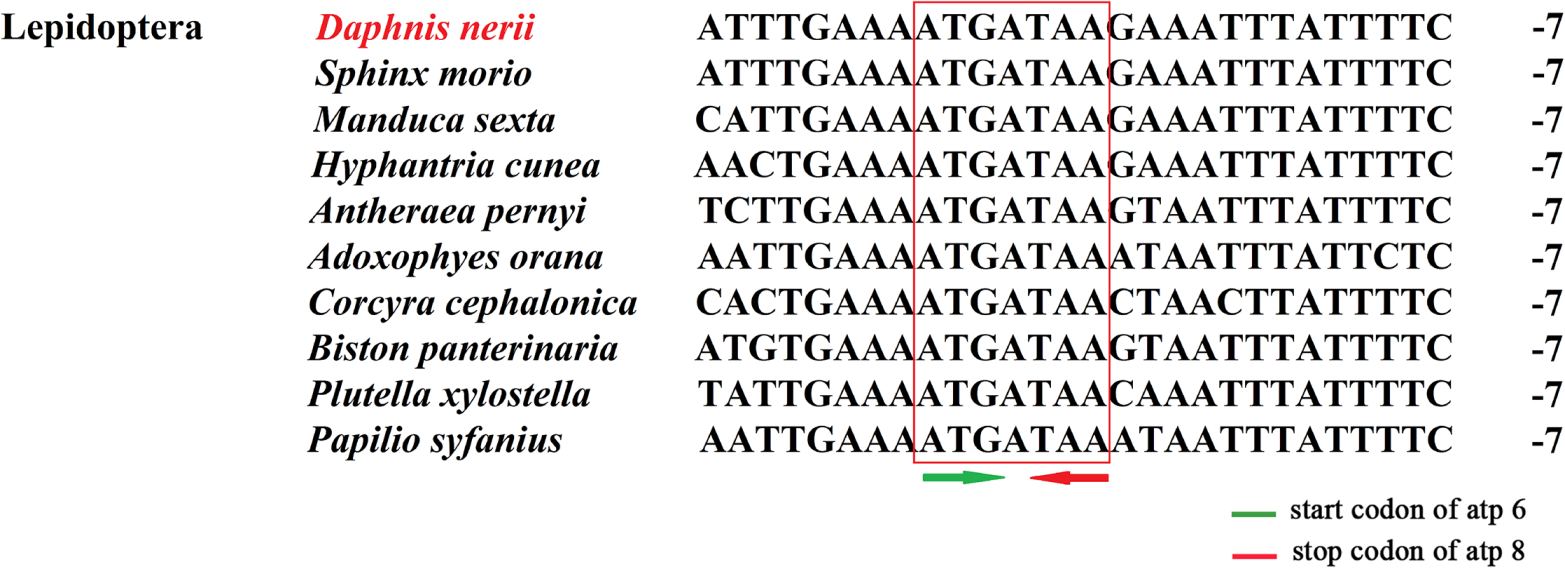
Alignment of overlapping region between *atp8* and *atp6* across Lepidoptera and other insects. The numbers on the right refer to intergenic nucleotides.

**Fig 7:**
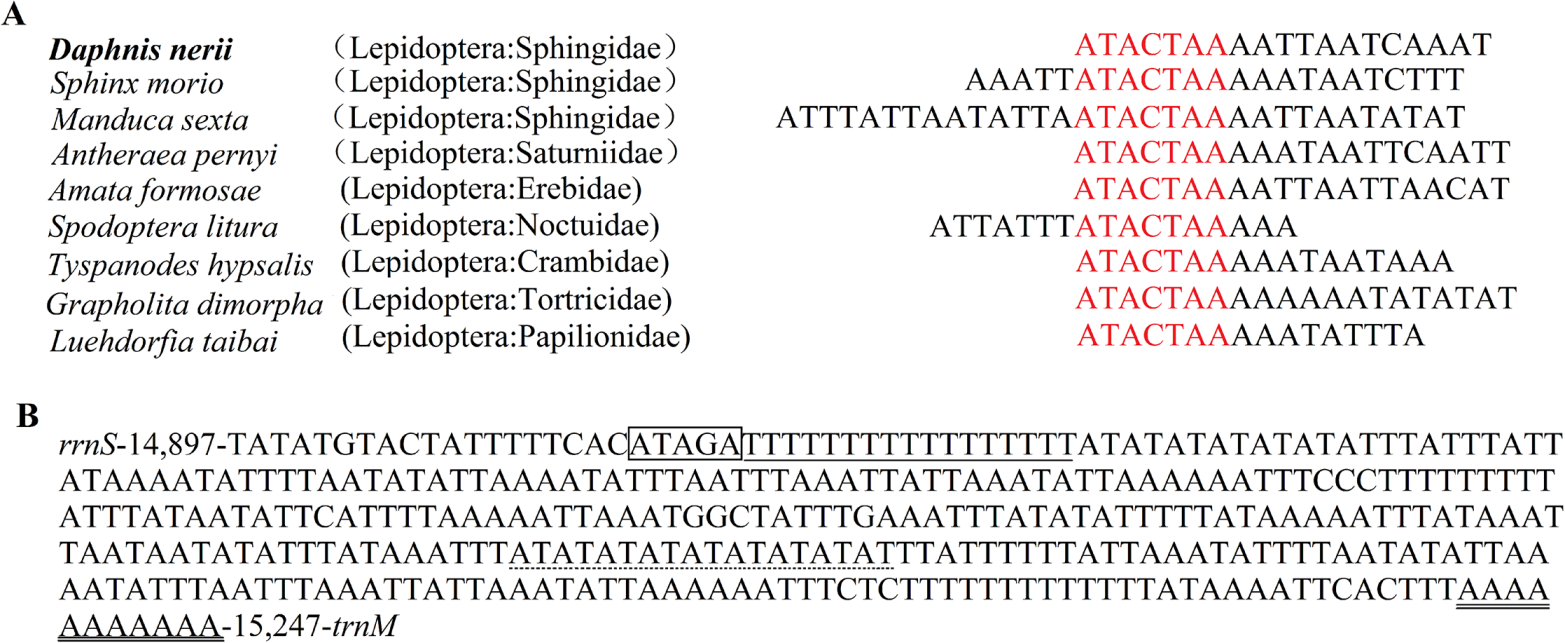
(A) Alignment of the intergenic spacer region between *trnS2* (UCN) and *nad1* of several Lepidopteran insects. The shaded ‘ATACTAA’ motif is conserved across the Lepidoptera order. (B) Features present in the A+T-rich region of *D*.*nerii*. The sequence is shown in the reverse strand. The ATATG motif is shaded. The poly-T stretch is underlined while the poly-A stretch is double underlined. The single microsatellite T/A repeats sequence are indicated by dotted underlining.

The mitogenome of *D*.*nerii* contains 12 overlapping regions with a total length of 26 bp. The six overlapping regions are resided between tRNA and tRNA (*trnW* and *trnC*, *trnA* and *trnR*, *trnD* and *trnS1*, *trnS1* and *trnE*, *trnE* and *trnF*, *trnT* and *trnP*), two between tRNA and protein (*nad2* and *trnW*, *trnS2* and *cytb*), and four between protein and protein (*atp6* and *atp8*, *atp6* and *cox3*, *nad4* and *nad4L*, *nad6* and *cytb*). The length of these sequences varies from 1 bp to 7 bp with the longest overlapping region present between *atp6* and *atp8* (Table 3), which is usually found in Lepidopteran mitogenomes[29, 30]. Further, we observed the longest region in ten Lepidopteran species (Fig 6), Which indicates the seven nucleotides sequence ATGATAA is a strikingly, common feature across Lepidopteran mitogenomes[6]. The mitogenome of *D*.*nerii* has 12 intergenic spacers in a total of 126 bp with a length varying in 1 to 55 bp. Of which there are four major intergenic spacers at least 10 bp in length (Table 3). The longest intergenic spacer (55bp) is located between the *trnQ* and *nad2*, with an extremely high A+T nucleotides content, this characteristic feature has been frequently described in Lepidopteran mitogenomes[21]. The 19 bp spacer between *trnS2* (UCN) and *nad1* contains the motif ATACTAA (Fig 7A) that is highly conserved region and found in most insect mtDNAs, and it seems to be a possible mitochondrial transcription termination peptide-binding site (mtTERM protein)[31].

### 3.5 The A+T-rich region

The A + T-rich region of *D*.*nerii* mitogenome is located between the *rrnS* and *trnM* with a length of 351 bp that is remarkably shorter than *G*. *dimorpha* (848 bp) and longer than *S*. *morio* (316 bp), but average when compared with that of other Lepidopteran mitogenomes and (Table 4). This region harbors the highest A+T content (95.16%) in the mtDNA, and most negative AT skewness (-0.126) and GC skewness (-0.413) (Table 4). We identified several short repeating sequences scattered throughout the entire region, including the motif ATAGA followed by a 17 bp poly-T stretch, a microsate-like (AT)_9_ element and a poly-A element upstream of *trnM* gene similar to other Lepidopteran mitogenomes (Fig 7B). The length of poly-T stretch varies from species to species[6, 20], while ATAGA region is conserved in Lepidoptera species[9].

### 3.6. Phylogenetic analyses

**Fig 8:**
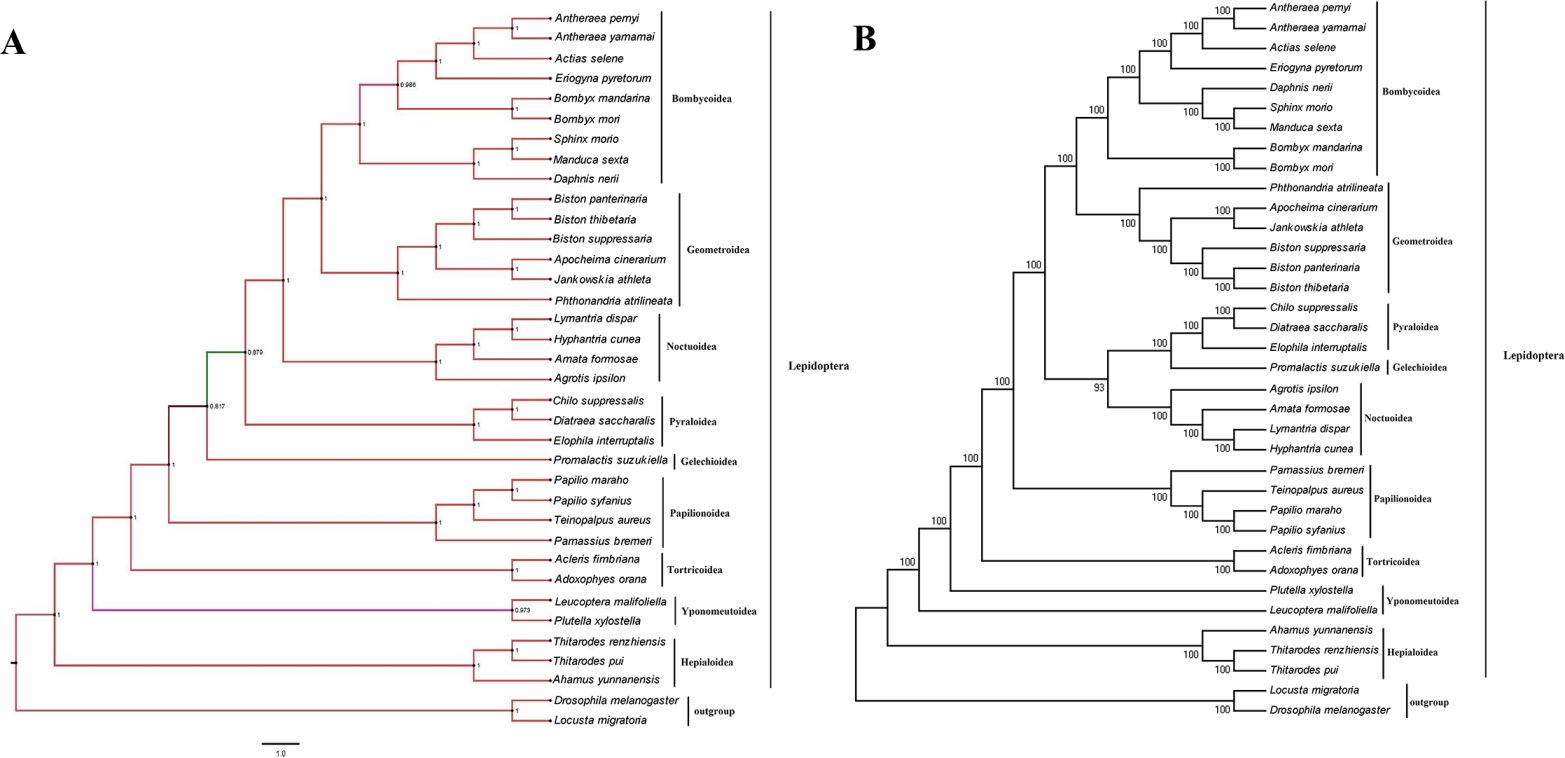
Tree showing the phylogenetic relationships among Lepidopteran insects, constructed using (A) Bayesian inference (BI). (B) Maximum Likelihood method (ML). Bootstrap values (1000 repetitions) of the branches are indicated. *Drosophila melanogaster* (U37541.1) and *Locustamigratoria* (NC_001712) were used as outgroups.

To reconstruct the phylogenetic relationship among Lepidopteran insects, the nucleotide sequences of the 13 PCGs were firstly aligned and then concatenated. The phylogenetic analyses showed that *D*.*nerii* has a close relationship to *M*. *sexta* and *S*. *morio* that was well supported from both BI and ML analyses (Fig 8A and 8B). The *D*. *nerii* is within the family Sphingidae (Bombycoidea) and clustered with other superfamilies, including the Geometroidea, Noctuoidea, Pyraloidea, Gelechioidea, Papilionoidea, Tortricoidea, Yponomeutoidea and Hepialoidea. Further the analyses revealed that Sphingidae is more closely related to Bombycidae than Saturniidae. Interestingly, Bombycoidea was more closely related to Noctuoideain ML methods, while in BI method Bombycoidea closely related to Geometroidea. These phylogenetic relationships are consistent with previously reportedstudies of Lepidopterans[11, 32]. We concluded from the present study that more research on the diverse Lepidoptera species is needed, to be able to understand better the relationships among them.

## Supporting information

S1 File Mitochondrial genome of *Daphnis nerii*. (SEQ)
